# 7-(4-Bromo­phen­yl)-9-phenyl-7*H*-pyrrolo[3,2-*e*]tetra­zolo[1,5-*c*]pyrimidine

**DOI:** 10.1107/S1600536810004368

**Published:** 2010-02-10

**Authors:** Mukesh M. Jotani, Rina D. Shah, Jerry P. Jasinski, Ray J. Butcher

**Affiliations:** a Department of Physics, Bhavan’s Sheth R.A. College of Science, Ahmedabad, Gujarat, 380 001, India; b Department of Chemistry, M.G. Science Institute, Navrangpura, Navrangpura, Ahmedabad, Gujarat, 380 009, India; c Department of Chemistry, Keene State College, 229 Main Street, Keene, NH 03435-2001, USA; d Department of Chemistry, Howard University, 525 College Street NW, Washington DC 20059, USA

## Abstract

In the title compound, C_18_H_11_BrN_6_, the phenyl ring is almost coplanar [dihedral angle 7.2 (1)°] with the planar (r.m.s. deviation 0.039 Å) tricyclic ring system while the 4-bromo­phenyl ring makes a dihedral angle of 33.98 (6)° with the ring system. Weak inter­molecular C—H⋯N and C—H⋯Br hydrogen-bonding inter­actions and π–π stacking [centroid–centroid distances = 3.7971 (17) and 3.5599 (16) Å] stabilize the crystal packing. A comparison of the structure to a MOPAC PM3 geometry optimization calculation *in vacuo* supports these observations.

## Related literature

For anti­cancer relationships, see: Hiedo & Yasuo (1960 ▶, 1961 ▶). For the synthesis of derivative compounds, see: Dave & Shukla (1997 ▶); Dave & Shah (1998 ▶). For graph-set motifs, see: Bernstein *et al.* (1995 ▶). For MOPAC PM3 calculations, see: Schmidt & Polik (2007 ▶).
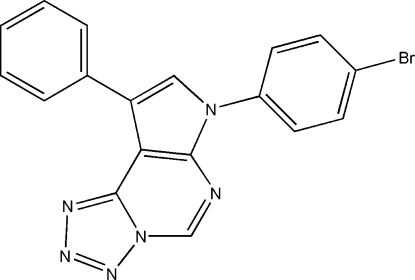


## Experimental

### 

#### Crystal data

C_18_H_11_BrN_6_
*M* *_r_* = 391.24
Monoclinic, 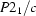

*a* = 12.0173 (5) Å
*b* = 17.4007 (7) Å
*c* = 7.4201 (4) Å
β = 91.004 (4)°
*V* = 1551.37 (12) Å^3^
*Z* = 4
Mo *K*α radiation
μ = 2.66 mm^−1^
*T* = 200 K
0.47 × 0.39 × 0.22 mm

#### Data collection

Oxford Diffraction Gemini diffractometer
Absorption correction: multi-scan (*CrysAlis RED*; Oxford Diffraction, 2007 ▶) *T*_min_ = 0.494, *T*_max_ = 1.000
13458 measured reflections
5032 independent reflections
2606 reflections with *I* > 2σ(*I*)
*R*_int_ = 0.050

#### Refinement

*R*[*F* ^2^ > 2σ(*F* ^2^)] = 0.036
*wR*(*F* ^2^) = 0.090
*S* = 1.14
5032 reflections
227 parameters
H-atom parameters constrained
Δρ_max_ = 0.72 e Å^−3^
Δρ_min_ = −0.71 e Å^−3^

### 

Data collection: *CrysAlis PRO* (Oxford Diffraction, 2007 ▶); cell refinement: *CrysAlis PRO*; data reduction: *CrysAlis PRO*; program(s) used to solve structure: *SHELXS97* (Sheldrick, 2008 ▶); program(s) used to refine structure: *SHELXL97* (Sheldrick, 2008 ▶); molecular graphics: *SHELXTL* (Sheldrick, 2008 ▶); software used to prepare material for publication: *SHELXTL*.

## Supplementary Material

Crystal structure: contains datablocks I. DOI: 10.1107/S1600536810004368/bt5188sup1.cif

Structure factors: contains datablocks I. DOI: 10.1107/S1600536810004368/bt5188Isup2.hkl

Additional supplementary materials:  crystallographic information; 3D view; checkCIF report

## Figures and Tables

**Table 1:** Hydrogen-bond geometry (Å, °)

*D*—H⋯*A*	*D*—H	H⋯*A*	*D*⋯*A*	*D*—H⋯*A*
C8—H8⋯N4^i^	0.95	2.54	3.421 (3)	154
C4—H4⋯N5^i^	0.95	2.60	3.532 (3)	166
C5—H5⋯Br^ii^	0.95	2.87	3.667 (3)	142

Symmetry codes: (i) 
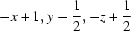
; (ii) 
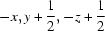
.

**Fig. 1.**
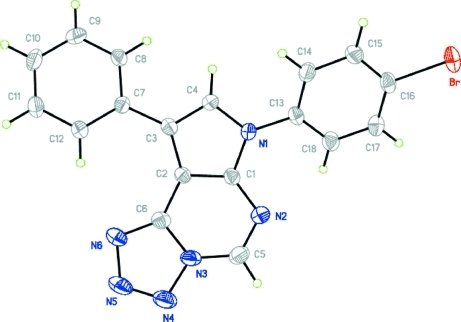
Molecular structure of C18H11BrN6, (I), showing the atom labeling scheme and 50% probability displacement ellipsoids.

**Fig. 2.**
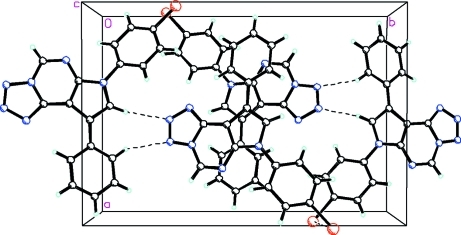
The molecular packing for (I) viewed down the c axis. Dashed lines indicate weak C—H···N intermolecular hydrogen bond interactions which link the molecule into chains propagating along the [011].

**Fig. 3.**
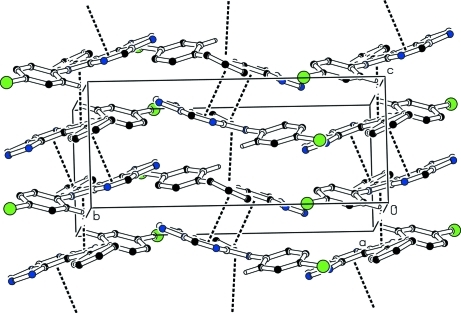
The molecular packing of (I), showing π—π stacking interactions along the c axis and forming a chain of molecules along [1 1 0].

## supplementary crystallographic information

### Comment

Fused tetrazoles are known to be related to a variety of biological activities including anticancer relationships (Hiedo & Yasuo, 1960, 1961). Moreover their reductive ring cleavage ability may result in the formation of synthetically important 4-aminopyrimidine derivatives which are building blocks for the construction of ring systems such as pyrimidine and imidazole (Dave & Shukla, 1997). Furthermore, nucleophilic substitution with NaN_3_ results in either the formation of an azido group or tetrazole ring. An example of these reactions has been the synthesis of (7-Bromophenyl)-9-phenyl 7*H*-[1,2,3,4]tetrazolo[1,5-c]pyrrolo[3,2-e] pyrimidine by two different routes, either from 4-chloro-5-phenyl-(7-Bromophenyl)-7*H*-pyrrolo[2,3-d]pyrimidine or from 4-hydrazino-5-phenyl-(7-Bromophenyl)-7*H*-pyrrolo[2,3-d]pyrimidine (Dave & Shah, 1998) and then reduced to 4-amino-5-phenyl-(7-Bromophenyl)-7*H*-pyrrolo[2,3-d]pyrimidine. In view of the importance of these findings, we report the crystal structure of the title compound, C_18_H_11_BrN_6_, (I).

The title compond, (I), contains a pyrrole ring with successively fused pyrimidine and tetrazole rings, together with bromophenyl and benzene rings bonded at the N1 and C3 positions in a nearly planar fashion (Fig. 1). The nearly planar conformation of the pyrimidine ring is significant in that it is often found to be puckered which may be affected by the tetrazole and pyrrole ring fusion here. The least squares planes of the pyrimidine and tetrazole rings are oriented at the angles of 2.62 (15)° and 3.15 (15)° with the best plane of the pyrrole ring, respectively. The ortho-substituted 4-bromophenyl ring is twisted considerably, whereas the benzene ring at C3 is almost co-planar with the mean plane of the pyrrole ring, each having dihedral angles of 32.62 (15)° and 9.97 (16)°, respectively.

The crystal sructure is supported by weak C—H···Br and C—H···N intermolecular and weak C—H···N intramolecular interactions which link the molecules into chains along the [011] (Fig. 2, Table 1). An R22(8) graph-set motif (Bernstein *et al.*, 1995) is established between the pyrrole, tetrazole and benzene rings resulting from these intermolecular interactions.

Crystal packing is also supported by two π-π stacking interactions (Fig. 3). One is between the centroids of two pyrrole rings [Cg2—Cg2; 3.7971 (17) Å; slippage = 1.318 Å; 1-x, 1-y, -z; Cg2 = N6/C3/C6/C5/C4]. The second is between the centroids of a pyrimidine (Cg3) and benzene (Cg4) ring [Cg3—Cg4: 3.5599 (16) Å; 1-x, 1-y, 1-z; Cg3 = N4/C1/C6/C3/N5/C2; Cg4 = C7—C12].

After a geometry optimized MOPAC PM3 computational calculation (Schmidt & Polik 2007) on (I), in vacuo, the angle between the mean planes of the pyrimidine, tetrazole and benzene groups become planar with the pyrrole ring in the local minimized structure. The dihedral angle between the ortho-substituted 4-bromophenyl ring and three planar tri-ring group becomes 42.28°. The separation of the H4A···H8A (2.111 Å) and H4A···H14A (2.301 Å) atoms between the pyrrole ring and the 4-bromophenyl and 9-phenyl rings before the calculations changed to 1.954 Å and 2.496 Å, respectively, after the calculation showing how the crystal packing effects significantly determine the twist of the 4-bromophenyl ring, in particular. In addition, the C3–C7 and N1–C13 bond lengths changed from 1.479 (3) Å and 1.430 (3) Å to 1.442 Å and 1.455 Å, respectively. It is clear that hydrogen bonding interactions and π-π stacking interactions significantly influence the twist angles for the molecule in this crystal.

### Experimental

The synthesis of (I) was carried out by two separate routes. Route 1: To a mixture of NaN_3_ (0.011 m), NH_4_Cl (0.011 m) and DMSO (20 ml) was added 4-chloro-5-phenyl-(7-bromophenyl)-7*H*-pyrrolo[2,3-d]pyrimidine (0.001 m) in portions and stirred for 2 hrs at 363 K to give the title compound. Route 2: 4-hydrazino-5-phenyl-(7-bromophenyl)-7*H*-pyrrolo[2,3-d]pyrimidine (0.01 m) was diazotized with an aqueous solution of NaNO_2_ (20% w/v, 4.2 ml) and glacial acetic acid (40 ml) at 273-278 K under stirring conditions for 2 hrs to give the title compound. Colorless, plate-like crystals of (I) were grown by slow evaporation from 1,4-dioxane solution.

### Refinement

All of the H atoms were placed in their calculated positions and then refined using the riding model with C—H = 0.95 Å, and with U_iso_(H) = 1.18-1.21U_eq_(C).

### Crystal data

**Table:**

C_18_H_11_BrN_6_	*F*(000) = 784
*M**_r_* = 391.24	*D*_x_ = 1.675 Mg m^−^^3^
Monoclinic, *P*2_1_/*c*	Mo *K*α radiation, λ = 0.71073 Å
Hall symbol: -P 2ybc	Cell parameters from 4168 reflections
*a* = 12.0173 (5) Å	θ = 4.8–32.4°
*b* = 17.4007 (7) Å	µ = 2.66 mm^−^^1^
*c* = 7.4201 (4) Å	*T* = 200 K
β = 91.004 (4)°	Plate, colorless
*V* = 1551.37 (12) Å^3^	0.47 × 0.39 × 0.22 mm
*Z* = 4	

### Data collection

**Table:**

Oxford Diffraction Gemini diffractometer	5032 independent reflections
Radiation source: fine-focus sealed tube	2606 reflections with *I* > 2σ(*I*)
graphite	*R*_int_ = 0.050
Detector resolution: 10.5081 pixels mm^-1^	θ_max_ = 32.5°, θ_min_ = 4.8°
φ and ω scans	*h* = −16→18
Absorption correction: multi-scan (*CrysAlis RED*; Oxford Diffraction, 2007)	*k* = −25→26
*T*_min_ = 0.494, *T*_max_ = 1.000	*l* = −11→11
13458 measured reflections	

### Refinement

**Table:**

Refinement on *F*^2^	Secondary atom site location: difference Fourier map
Least-squares matrix: full	Hydrogen site location: inferred from neighbouring sites
*R*[*F*^2^ > 2σ(*F*^2^)] = 0.036	H-atom parameters constrained
*wR*(*F*^2^) = 0.090	*w* = 1/[σ^2^(*F*_o_^2^) + (0.0295*P*)^2^] where *P* = (*F*_o_^2^ + 2*F*_c_^2^)/3
*S* = 1.14	(Δ/σ)_max_ < 0.001
5032 reflections	Δρ_max_ = 0.72 e Å^−^^3^
227 parameters	Δρ_min_ = −0.71 e Å^−^^3^
0 restraints	Extinction correction: *SHELXL*, Fc^*^=kFc[1+0.001xFc^2^λ^3^/sin(2θ)]^-1/4^
Primary atom site location: structure-invariant direct methods	Extinction coefficient: 0.0033 (4)

### Special details

**Table:**

Experimental. (CrysAlis RED; Oxford Diffraction, 2007)
Geometry. All esds (except the esd in the dihedral angle between two l.s. planes) are estimated using the full covariance matrix. The cell esds are taken into account individually in the estimation of esds in distances, angles and torsion angles; correlations between esds in cell parameters are only used when they are defined by crystal symmetry. An approximate (isotropic) treatment of cell esds is used for estimating esds involving l.s. planes.
Refinement. Refinement of *F*^2^ against ALL reflections. The weighted *R*-factor *wR* and goodness of fit *S* are based on *F*^2^, conventional *R*-factors *R* are based on *F*, with *F* set to zero for negative *F*^2^. The threshold expression of *F*^2^ > σ(*F*^2^) is used only for calculating *R*-factors(gt) *etc*. and is not relevant to the choice of reflections for refinement. *R*-factors based on *F*^2^ are statistically about twice as large as those based on *F*, and *R*- factors based on ALL data will be even larger.

### Fractional atomic coordinates and isotropic or equivalent isotropic displacement parameters (Å^2^)

**Table:**

	*x*	*y*	*z*	*U*_iso_*/*U*_eq_	
Br	−0.01667 (2)	0.226033 (17)	0.00063 (4)	0.03758 (11)	
N1	0.35315 (14)	0.45391 (11)	0.1749 (3)	0.0245 (5)	
N2	0.24838 (15)	0.57000 (12)	0.2186 (3)	0.0300 (5)	
N3	0.35441 (16)	0.67017 (11)	0.3359 (3)	0.0292 (5)	
N4	0.3685 (2)	0.74165 (13)	0.4080 (3)	0.0406 (6)	
N5	0.4737 (2)	0.74487 (13)	0.4525 (4)	0.0432 (7)	
N6	0.52928 (17)	0.67915 (12)	0.4136 (3)	0.0359 (6)	
C1	0.34572 (18)	0.52985 (14)	0.2224 (3)	0.0235 (5)	
C2	0.45169 (18)	0.55548 (13)	0.2756 (3)	0.0219 (5)	
C3	0.52682 (19)	0.49222 (13)	0.2569 (3)	0.0230 (5)	
C4	0.46260 (17)	0.43241 (14)	0.1955 (4)	0.0245 (6)	
H4	0.4902	0.3825	0.1703	0.029*	
C5	0.2555 (2)	0.63991 (16)	0.2741 (4)	0.0338 (7)	
H5	0.1908	0.6713	0.2722	0.041*	
C6	0.45368 (19)	0.63216 (14)	0.3404 (4)	0.0263 (6)	
C7	0.64817 (18)	0.48691 (13)	0.2913 (3)	0.0225 (5)	
C8	0.70547 (19)	0.42147 (14)	0.2367 (4)	0.0277 (6)	
H8	0.6659	0.3814	0.1769	0.033*	
C9	0.8188 (2)	0.41373 (15)	0.2680 (4)	0.0344 (7)	
H9	0.8560	0.3687	0.2289	0.041*	
C10	0.8778 (2)	0.47079 (17)	0.3551 (4)	0.0351 (7)	
H10	0.9556	0.4654	0.3767	0.042*	
C11	0.82296 (19)	0.53569 (16)	0.4107 (4)	0.0344 (7)	
H11	0.8634	0.5752	0.4713	0.041*	
C12	0.70894 (18)	0.54451 (14)	0.3794 (4)	0.0283 (6)	
H12	0.6725	0.5899	0.4181	0.034*	
C13	0.26457 (18)	0.40147 (14)	0.1343 (3)	0.0238 (6)	
C14	0.16953 (19)	0.42519 (15)	0.0414 (4)	0.0301 (6)	
H14	0.1623	0.4771	0.0035	0.036*	
C15	0.08552 (19)	0.37323 (16)	0.0039 (4)	0.0308 (6)	
H15	0.0195	0.3894	−0.0574	0.037*	
C16	0.09811 (19)	0.29777 (15)	0.0561 (4)	0.0282 (6)	
C17	0.19254 (19)	0.27285 (15)	0.1480 (4)	0.0294 (6)	
H17	0.2005	0.2205	0.1822	0.035*	
C18	0.27530 (18)	0.32577 (15)	0.1893 (4)	0.0293 (6)	
H18	0.3397	0.3100	0.2556	0.035*	

### Atomic displacement parameters (Å^2^)

**Table:**

	*U*^11^	*U*^22^	*U*^33^	*U*^12^	*U*^13^	*U*^23^
Br	0.03221 (15)	0.04452 (19)	0.03605 (18)	−0.01798 (12)	0.00207 (11)	−0.00499 (15)
N1	0.0192 (9)	0.0240 (11)	0.0302 (13)	−0.0015 (8)	−0.0006 (9)	−0.0010 (10)
N2	0.0265 (11)	0.0277 (12)	0.0361 (15)	0.0033 (9)	0.0021 (10)	−0.0018 (11)
N3	0.0332 (11)	0.0193 (11)	0.0354 (14)	0.0034 (9)	0.0050 (10)	−0.0002 (10)
N4	0.0478 (14)	0.0232 (13)	0.0509 (17)	0.0014 (10)	0.0049 (12)	−0.0054 (12)
N5	0.0527 (15)	0.0195 (12)	0.057 (2)	−0.0027 (10)	0.0016 (13)	−0.0071 (12)
N6	0.0398 (12)	0.0205 (12)	0.0473 (16)	−0.0030 (9)	−0.0007 (11)	−0.0038 (11)
C1	0.0236 (11)	0.0220 (12)	0.0250 (15)	−0.0031 (10)	0.0021 (10)	−0.0002 (11)
C2	0.0249 (12)	0.0186 (12)	0.0222 (15)	−0.0019 (9)	0.0009 (10)	0.0026 (11)
C3	0.0279 (13)	0.0211 (13)	0.0199 (13)	−0.0029 (9)	−0.0028 (10)	0.0028 (11)
C4	0.0215 (11)	0.0226 (13)	0.0295 (16)	0.0015 (9)	0.0001 (10)	−0.0001 (12)
C5	0.0281 (13)	0.0334 (16)	0.0399 (18)	0.0067 (11)	0.0029 (12)	−0.0003 (14)
C6	0.0299 (13)	0.0198 (13)	0.0294 (16)	−0.0005 (10)	0.0044 (11)	0.0061 (12)
C7	0.0206 (12)	0.0232 (13)	0.0237 (14)	−0.0020 (9)	−0.0001 (10)	0.0048 (11)
C8	0.0274 (12)	0.0210 (13)	0.0343 (17)	−0.0041 (10)	−0.0050 (11)	0.0037 (12)
C9	0.0342 (14)	0.0269 (15)	0.0418 (19)	0.0075 (11)	−0.0035 (13)	0.0054 (13)
C10	0.0237 (13)	0.0426 (16)	0.0388 (19)	0.0004 (12)	−0.0085 (12)	0.0022 (14)
C11	0.0291 (13)	0.0352 (15)	0.0387 (19)	−0.0065 (12)	−0.0077 (12)	−0.0027 (14)
C12	0.0261 (12)	0.0242 (14)	0.0346 (17)	−0.0022 (10)	−0.0022 (11)	−0.0011 (12)
C13	0.0243 (12)	0.0256 (13)	0.0216 (15)	−0.0063 (10)	0.0034 (10)	−0.0033 (11)
C14	0.0275 (12)	0.0294 (14)	0.0334 (17)	−0.0039 (10)	0.0003 (12)	0.0052 (13)
C15	0.0251 (13)	0.0369 (16)	0.0304 (16)	−0.0043 (11)	−0.0025 (11)	0.0022 (13)
C16	0.0255 (12)	0.0331 (15)	0.0263 (16)	−0.0093 (10)	0.0075 (11)	−0.0060 (12)
C17	0.0291 (13)	0.0252 (13)	0.0341 (16)	−0.0027 (11)	0.0036 (11)	0.0019 (13)
C18	0.0213 (12)	0.0313 (15)	0.0352 (17)	−0.0008 (10)	−0.0010 (11)	0.0036 (13)

### Geometric parameters (Å, °)

**Table:**

Br—C16	1.900 (2)	C7—C12	1.396 (3)
N1—C1	1.371 (3)	C8—C9	1.383 (3)
N1—C4	1.373 (3)	C8—H8	0.9500
N1—C13	1.430 (3)	C9—C10	1.375 (4)
N2—C5	1.287 (3)	C9—H9	0.9500
N2—C1	1.362 (3)	C10—C11	1.374 (4)
N3—N4	1.363 (3)	C10—H10	0.9500
N3—C6	1.364 (3)	C11—C12	1.394 (3)
N3—C5	1.371 (3)	C11—H11	0.9500
N4—N5	1.301 (3)	C12—H12	0.9500
N5—N6	1.358 (3)	C13—C18	1.384 (3)
N6—C6	1.331 (3)	C13—C14	1.387 (3)
C1—C2	1.400 (3)	C14—C15	1.380 (3)
C2—C6	1.418 (3)	C14—H14	0.9500
C2—C3	1.432 (3)	C15—C16	1.377 (4)
C3—C4	1.369 (3)	C15—H15	0.9500
C3—C7	1.479 (3)	C16—C17	1.383 (3)
C4—H4	0.9500	C17—C18	1.386 (3)
C5—H5	0.9500	C17—H17	0.9500
C7—C8	1.394 (3)	C18—H18	0.9500
			
C1—N1—C4	107.48 (18)	C9—C8—H8	119.3
C1—N1—C13	128.14 (18)	C7—C8—H8	119.3
C4—N1—C13	123.9 (2)	C10—C9—C8	120.4 (2)
C5—N2—C1	115.2 (2)	C10—C9—H9	119.8
N4—N3—C6	109.3 (2)	C8—C9—H9	119.8
N4—N3—C5	125.7 (2)	C11—C10—C9	119.2 (2)
C6—N3—C5	125.1 (2)	C11—C10—H10	120.4
N5—N4—N3	104.6 (2)	C9—C10—H10	120.4
N4—N5—N6	112.9 (2)	C10—C11—C12	121.0 (2)
C6—N6—N5	105.6 (2)	C10—C11—H11	119.5
N2—C1—N1	123.28 (19)	C12—C11—H11	119.5
N2—C1—C2	128.2 (2)	C11—C12—C7	120.2 (2)
N1—C1—C2	108.47 (19)	C11—C12—H12	119.9
C1—C2—C6	113.9 (2)	C7—C12—H12	119.9
C1—C2—C3	107.4 (2)	C18—C13—C14	120.1 (2)
C6—C2—C3	138.5 (2)	C18—C13—N1	118.7 (2)
C4—C3—C2	105.3 (2)	C14—C13—N1	121.2 (2)
C4—C3—C7	123.9 (2)	C15—C14—C13	119.9 (2)
C2—C3—C7	130.7 (2)	C15—C14—H14	120.1
C3—C4—N1	111.3 (2)	C13—C14—H14	120.1
C3—C4—H4	124.3	C14—C15—C16	119.5 (2)
N1—C4—H4	124.3	C14—C15—H15	120.3
N2—C5—N3	121.5 (2)	C16—C15—H15	120.3
N2—C5—H5	119.2	C15—C16—C17	121.5 (2)
N3—C5—H5	119.2	C15—C16—Br	119.33 (19)
N6—C6—N3	107.6 (2)	C17—C16—Br	119.1 (2)
N6—C6—C2	136.3 (2)	C16—C17—C18	118.7 (2)
N3—C6—C2	116.0 (2)	C16—C17—H17	120.7
C8—C7—C12	117.7 (2)	C18—C17—H17	120.7
C8—C7—C3	119.4 (2)	C17—C18—C13	120.3 (2)
C12—C7—C3	122.8 (2)	C17—C18—H18	119.9
C9—C8—C7	121.4 (2)	C13—C18—H18	119.9
			
C6—N3—N4—N5	0.2 (3)	C1—C2—C6—N6	175.2 (3)
C5—N3—N4—N5	178.1 (3)	C3—C2—C6—N6	0.1 (6)
N3—N4—N5—N6	−0.1 (3)	C1—C2—C6—N3	−2.1 (3)
N4—N5—N6—C6	−0.1 (3)	C3—C2—C6—N3	−177.2 (3)
C5—N2—C1—N1	177.5 (2)	C4—C3—C7—C8	9.1 (4)
C5—N2—C1—C2	−1.3 (4)	C2—C3—C7—C8	−170.4 (3)
C4—N1—C1—N2	−179.8 (2)	C4—C3—C7—C12	−169.9 (3)
C13—N1—C1—N2	−7.7 (4)	C2—C3—C7—C12	10.6 (4)
C4—N1—C1—C2	−0.8 (3)	C12—C7—C8—C9	−0.2 (4)
C13—N1—C1—C2	171.3 (2)	C3—C7—C8—C9	−179.3 (2)
N2—C1—C2—C6	3.1 (4)	C7—C8—C9—C10	0.3 (4)
N1—C1—C2—C6	−175.8 (2)	C8—C9—C10—C11	−0.1 (4)
N2—C1—C2—C3	179.7 (2)	C9—C10—C11—C12	−0.2 (4)
N1—C1—C2—C3	0.8 (3)	C10—C11—C12—C7	0.3 (4)
C1—C2—C3—C4	−0.5 (3)	C8—C7—C12—C11	−0.1 (4)
C6—C2—C3—C4	174.9 (3)	C3—C7—C12—C11	178.9 (2)
C1—C2—C3—C7	179.1 (2)	C1—N1—C13—C18	−143.0 (3)
C6—C2—C3—C7	−5.6 (5)	C4—N1—C13—C18	27.9 (4)
C2—C3—C4—N1	0.0 (3)	C1—N1—C13—C14	37.2 (4)
C7—C3—C4—N1	−179.6 (2)	C4—N1—C13—C14	−151.9 (2)
C1—N1—C4—C3	0.5 (3)	C18—C13—C14—C15	0.1 (4)
C13—N1—C4—C3	−172.0 (2)	N1—C13—C14—C15	179.9 (2)
C1—N2—C5—N3	−1.4 (4)	C13—C14—C15—C16	−1.4 (4)
N4—N3—C5—N2	−175.5 (3)	C14—C15—C16—C17	1.0 (4)
C6—N3—C5—N2	2.1 (4)	C14—C15—C16—Br	−178.9 (2)
N5—N6—C6—N3	0.2 (3)	C15—C16—C17—C18	0.7 (4)
N5—N6—C6—C2	−177.2 (3)	Br—C16—C17—C18	−179.4 (2)
N4—N3—C6—N6	−0.3 (3)	C16—C17—C18—C13	−2.0 (4)
C5—N3—C6—N6	−178.2 (2)	C14—C13—C18—C17	1.6 (4)
N4—N3—C6—C2	177.8 (2)	N1—C13—C18—C17	−178.2 (2)
C5—N3—C6—C2	−0.1 (4)		

### Hydrogen-bond geometry (Å, °)

**Table:**

*D*—H···*A*	*D*—H	H···*A*	*D*···*A*	*D*—H···*A*
C8—H8···N4^i^	0.95	2.54	3.421 (3)	154
C4—H4···N5^i^	0.95	2.60	3.532 (3)	166
C5—H5···Br^ii^	0.95	2.87	3.667 (3)	142

Symmetry codes: (i) −*x*+1, *y*−1/2, −*z*+1/2; (ii) −*x*, *y*+1/2, −*z*+1/2.

## References

[bb1] Bernstein, J., Davis, R. E., Shimoni, L. & Chang, N. L. (1995). *Angew. Chem. Int. Ed. Engl.* **34**, 1555–1573.

[bb2] Dave, C. G. & Shah, R. D. (1998). *J. Heterocycl. Chem.* **35**, 1295–1300.

[bb3] Dave, C. G. & Shukla, M. C. (1997). *J. Heterocycl. Chem.* **34**, 1805–1808.

[bb4] Hiedo, K. & Yasuo, M. (1960). Japan Patent 17,236.

[bb5] Hiedo, K. & Yasuo, M. (1961). *Chem. Abstr.* **55**, 17664.

[bb6] Oxford Diffraction (2007). *CrysAlis PRO* and *CrysAlis RED* Oxford Diffraction Ltd, Abingdon, England.

[bb7] Schmidt, J. R. & Polik, W. F. (2007). *WebMO Pro* WebMO, LLC: Holland, MI, USA, available from http://www.webmo.net.

[bb8] Sheldrick, G. M. (2008). *Acta Cryst.* A**64**, 112–122. 10.1107/S0108767307043930 18156677

